# Hospital Wastewater as a Reservoir of Contaminants of Emerging Concern: A Study Report from South America, Chile

**DOI:** 10.3390/antibiotics14111111

**Published:** 2025-11-04

**Authors:** Eduardo J. Aguilar-Rangel, Francisca Paredes-Cárcamo, Maikol D. Andrade, Danilo Contreras-Sánchez, Vanessa Rain-Medina, Javier Campanini-Salinas, Daniel A. Medina

**Affiliations:** 1 Escuela Nacional de Estudios Superiores Unidad Morelia, Universidad Nacional Autónoma de México, Morelia 58190, Mexico; edaguilar@ciencias.unam.mx; 2 Laboratorio de Microbiómica, Escuela Nacional de Estudios Superiores Unidad Morelia, Universidad Nacional Autónoma de México, Morelia 58190, Mexico; 3 Laboratorio Institucional, Universidad San Sebastián, Puerto Montt 5501842, Chile; fparedesc5@correo.uss.cl (F.P.-C.); mdomihuala@correo.uss.cl (M.D.A.); vrainm@correo.uss.cl (V.R.-M.);; 4 Escuela de Química y Farmacia, Facultad de Ciencias, Universidad San Sebastián, Puerto Montt 5501842, Chile

**Keywords:** hospital wastewater, antimicrobial resistance, metagenomic analysis

## Abstract

**Background/Objectives:** Hospital wastewater is a complex effluent containing a wide range of biological and chemical contaminants, including pharmaceuticals, pathogens, and antimicrobial resistance determinants. These discharges pose a growing threat to aquatic ecosystems and public health, particularly in regions where wastewater treatment is insufficient. This study aimed to characterize the chemical and microbiological composition of untreated effluent from a tertiary care hospital in southern Chile, focusing on contaminants of emerging concern. **Methods:** Wastewater samples were collected at the hospital outlet before any treatment. The presence of two commonly used pharmaceutical compounds, paracetamol and amoxicillin, was quantified using high-performance liquid chromatography (HPLC). Bacterial isolation was performed using selective media, and antibiotic susceptibility testing was conducted via the disk diffusion method following CLSI guidelines. In addition, metagenomic DNA was extracted and sequenced to assess microbial community composition and functional gene content, focusing on the identification of resistance genes and potential pathogens. **Results:** A total of 42 bacterial isolates were recovered, including genera with known pathogenic potential such as *Aeromonas*, *Klebsiella*, and *Enterococcus*. Antibiotic susceptibility tests revealed a high prevalence of multidrug-resistant strains. Metagenomic analysis identified the dominance of Bacillota and Bacteroidota, together with 56 antimicrobial-resistance gene (ARG) families and 38 virulence-factor families. Functional gene analysis indicated the presence of efflux-pump systems, β-lactamases, and mobile genetic elements, suggesting that untreated hospital effluents serve as potential sources of resistance and virulence determinants entering the environment. Paracetamol was detected in all samples, with an average concentration of 277.4 ± 10.7 µg/L; amoxicillin was not detected, likely due to its instability and rapid degradation in the wastewater matrix. **Conclusions:** These findings highlight the complex microbiological and chemical burden of untreated hospital wastewater and reinforce the need for continuous monitoring and improved treatment strategies to mitigate environmental dissemination of antibiotic resistance.

## 1. Introduction

Water pollution is an increasingly serious issue, accentuated by the loss of freshwater bodies and the overexploitation of these resources [1]. It is currently recognized as a major threat to the health of the environment, animals, and humans. However, the degree of impact caused by water pollution varies in each case, depending on the source of contamination, which determines both its quantity and quality, and consequently, its effect on the surrounding environment and human health [2]. Among the different sources of environmental pollution, wastewater discharges stand out as particularly significant due to their abundance, the diversity of their contaminants, and the insufficient waste management policies in many developing countries, including Chile. Wastewater properties depend on the origin of the pollutants, such as domestic, agricultural, industrial, and hospital sources [3]. Among these, hospital wastewater is particularly significant due to its high volume and the wide variety of substances present in the effluents, which originate from internal sources such as analysis laboratories, operating rooms, laundry, and restrooms [4,5]. The composition of hospital wastewater includes a solid fraction, such as discarded instruments, laboratory supplies, and even human waste, and a liquid fraction dominated by a significant biological and chemical load, which is considered more hazardous.

In general, the liquid fraction of hospital wastewater contains a high load of pharmaceuticals (including hormones, antibiotics, anti-inflammatory agents, and analgesic drugs), along with organic compounds, disinfectants, pathogens, and even radioactive substances [6,7,8]. Many of these compounds have been identified as having considerable ecotoxicological potential, with mutagenic and carcinogenic effects on human health. On the other hand, the presence of antibiotics is directly correlated with the rise in resistance mechanisms and their dissemination in the environment [6,9,10]. Moreover, the conditions in wastewater systems favor the proliferation and spread of pathogenic microorganisms. The most common diseases associated with wastewater discharge and transport include intestinal, respiratory, and renal infections, among others. Hospital wastewater has emerged as a critical environmental concern due to its complex composition of pharmaceuticals, antibiotic residues, resistant microorganisms, and mobile genetic elements. Unlike domestic effluents, hospital discharges contain disproportionately high loads of antimicrobials and pathogens, including multidrug-resistant bacteria such as ESBL-producing Enterobacteriaceae and carbapenem-resistant strains, which can disseminate resistance genes into aquatic ecosystems [6,8]. Several studies have shown that common wastewater treatment plants are not specifically designed to remove hospital-related contaminants, allowing antibiotic residues, heavy metals, and resistant bacteria to persist and create favorable conditions for horizontal gene transfer [11,12]. This situation is particularly problematic in regions where hospital effluents are discharged untreated into municipal sewers, contributing significantly to the environmental spread of antimicrobial resistance [8,13]. Recent reviews highlight that the presence of clinically relevant genera such as *Klebsiella*, *Acinetobacter*, *Enterococcus*, and *Pseudomonas* in hospital effluents represents a direct risk to human health, as these organisms frequently harbor critical resistance genes, including *bla*NDM, *bla*KPC, and mcr-1 [8,10,11]. Moreover, residues of widely used drugs such as oxytetracyclines, fluoroquinolones, and β-lactams have been detected at concerning levels, with potential downstream impacts on aquatic organisms and even seafood destined for human consumption [6]. Advanced treatment technologies, including ozonation and UV-based disinfection, have been proposed as effective measures to reduce the microbial and chemical burden of these effluents, achieving over 99% reduction in resistant bacteria in pilot studies [3,14,15,16,17]. Nevertheless, the persistence of resistance determinants in treated effluents underscores the urgent need for integrated surveillance and the inclusion of hospital wastewater in national and international AMR mitigation policies [11]. Collectively, these findings illustrate how hospital effluents serve as hotspots for the amplification and dissemination of resistance, positioning them at the frontline of the global AMR crisis.

The growing concern arises from the fact that current treatment systems for hospital wastewater are often inadequate or, in some cases, entirely lacking. As a result, hospital effluents are frequently discharged into municipal sewage networks or directly released into natural ecosystems, including rivers, lakes, and other aquatic environments, where they may contribute to the spread of contaminants of emerging concern. These practices not only pose significant risks to human health but also contribute to serious environmental problems, including contamination and the disruption of entire ecosystems [2,5]. Among the primary concerns are the negative impacts on aquatic organisms and the degradation of ecosystem services. Consequently, the management of hospital wastewater has recently emerged as a critical issue, driven by challenges related to its accumulation, environmental persistence, and potential adverse effects on both ecological systems and public health. Various strategies have been proposed to address this growing concern, with wastewater treatment being the most implemented and effective approach to date [6,15,18].

In the present study, we evaluated the biological and chemical characteristics of wastewater effluent from a hospital in southern Chile. The objectives were to characterize microbial isolates, the resistome, genetic elements, potential pathogens, and pharmaceutical compounds present in the hospital’s untreated effluent. We hypothesize that hospital wastewater contains a high load of microorganisms with pathogenic potential and a wide diversity of resistance mechanisms, which constitute emerging pollutants that are not monitored in current Chilean regulations. This study represents an exploratory effort aimed at elucidating the microbiological and molecular complexity of hospital wastewater as a potential reservoir of emerging contaminants in southern Chile. To achieve this, we combined classical microbiological approaches (including bacterial isolation and antibiotic susceptibility testing) with microbial genomic analyses based on shotgun metagenomics and functional annotation of resistance and virulence determinants. This integrative framework provides a comprehensive overview of the microbial communities and genetic elements present in hospital effluents, enabling the identification of potential public health risks and environmental impacts under the One Health perspective. By merging culture-dependent and culture-independent methodologies, this work contributes to establishing a baseline for future surveillance and management strategies of emerging contaminants in healthcare-related aquatic systems. To our knowledge, this is the first metagenomic assessment of hospital wastewater in southern Chile combining resistome and virulome profiling with chemical and microbiological characterization.

## 2. Results

### 2.1. Microbial Pathogens and Virulence Potential in Wastewater

Hospital wastewater revealed a heterogeneous community of clinically and environmentally relevant bacteria, dominated by Gram-negative isolates. The genus *Aeromonas* was the most frequent isolate identified, with representatives including *A. allosaccharophila*, *A. hydrophila*, *A. salmonicida*, *A. rivipollensis*, and additional *Aeromonas* spp. These organisms, although typically aquatic, are recognized as opportunistic pathogens and potential carriers of mobile resistance elements. Other Gram-negative bacteria of concern included *Escherichia coli* and *Pseudomonas* spp., both classical indicators of fecal contamination and known for their intrinsic and acquired multidrug resistance, particularly against β-lactams and fluoroquinolones. Multiple isolates of *Klebsiella pneumoniae* and one *Raoultella ornithinolytica* were also identified; both are clinically significant Enterobacteriaceae associated with nosocomial infections and the carriage of extended-spectrum β-lactamases (ESBLs) and carbapenemases. The recovery of several *K. pneumoniae* isolates in a single effluent highlights the continuous release of high-risk clones into the environment. The only Gram-positive isolate was *Enterococcus faecium*, a species considered a critical nosocomial pathogen due to its ability to develop resistance to vancomycin and other last-resort antimicrobials. Its presence in hospital effluent suggests that wastewater acts as a reservoir not only for multidrug-resistant Gram-negative bacteria but also for highly resilient Gram-positive pathogens (Table 1). Interestingly, the presence of the 14 microbial isolates was further identified by taxonomic analysis using shotgun metagenomics, in which the bacteria of the genera *Bacteroides, Faecalibacterium, Acidovorax, Pseudomonas, Blautia, Collinsella, Alistipes, Acinetobacter, Segatella, Parabacteroides,* and *Enterobacter*, were the top 10 most prevalent in the samples analyzed (Supplementary File S1). Overall, the bacterial profile underscores the dual role of hospital wastewater as both a reflection of patient-associated microbiota and a conduit for the environmental dissemination of bacteria. These findings highlight the urgent need for systematic monitoring and treatment strategies to mitigate the release of pathogens into surrounding ecosystems.

A metagenomic survey based on a virulence factor database was carried out to identify genes associated with pathogenic potential and their corresponding bacterial hosts (Table 2, Supplementary File S1). The metagenomic assemblies obtained from the August and October hospital wastewater samples showed overall good quality metrics, supporting reliable downstream taxonomic and functional analyses. For the August dataset, the total assembly length reached 0.42 Gb, with an N50 of 1276 bp, an L50 of 67,216 contigs, GC 48.5%, and a longest contig of 245,340 bp. In comparison, the October assembly resulted in a slightly smaller total length of 0.37 Gb, with an N50 of 1446 bp, an L50 of 50,912 contigs, GC 46.2%, and a maximum contig length of 401,227 bp. Although both datasets differed slightly, the assembly metrics were consistent with typical metagenomic results obtained from complex environmental matrices [19,20]. The taxonomic profile revealed the predominance of Bacillota (Firmicutes) and Bacteroidota (Supplementary File 1), followed by Pseudomonadota and Actinomycetota (Actinobacteriota). Gene screening revealed the presence of potential pathogens according to their genetic content, with a predominance of motility-related genes such as those from the *flg*, *fli*, and *pil* gene families. Additionally, genes involved in the alginate biosynthesis pathway (alg family) were notably abundant in the August samples but were absent in those collected in October. Overall, *Pseudomonas aeruginosa* harbored the highest number of pathogenicity-related genes. However, molecular markers associated with pathogenic potential were also detected in *Aeromonas hydrophyla*, *Aeromonas salmonicida*, *Acitenobacter baumannii*, *Klebsiella pneumoniae,* and in less presence on *Escherichia coli*, *Salmonella enterica*, *Neisseria meningitidis*, *Brucella melitensis*, and *Shigella dysenteriae* (Supplementary File S1).

### 2.2. Antimicrobial Resistances in Wastewater

**Figure 1:**
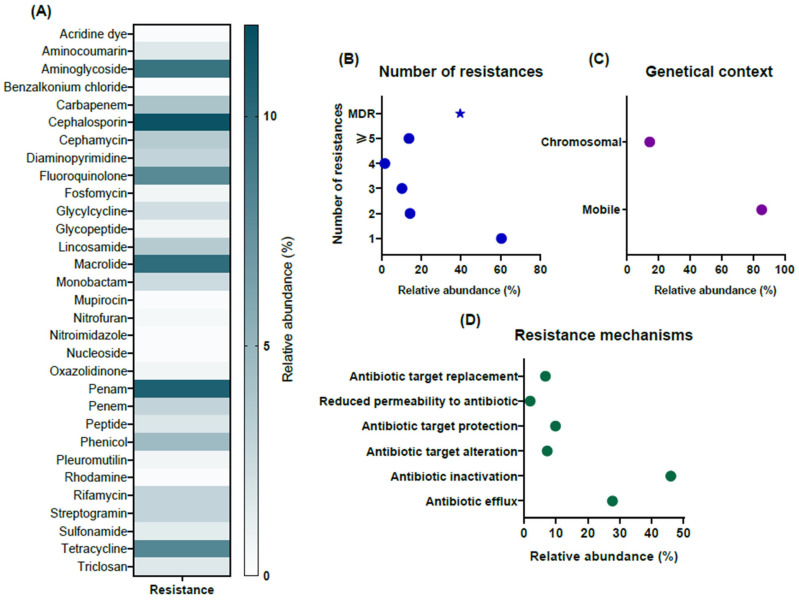
Antimicrobial resistance genes (ARGs) detected in hospital wastewater by metagenomic screening. (**A**) Distribution of ARGs across 31 antimicrobial families, with cephalosporins, penams, macrolides, aminoglycosides, tetracyclines, and fluoroquinolones being the most abundant. (**B**) Multidrug resistance profile showing that ~40% of ARGs confer resistance to multiple drug classes, with 14% linked to five or more. (**C**) Genetic context of ARGs indicating their predominant association with mobile genetic elements such as plasmids and integrons, in addition to chromosomal DNA. (**D**) Functional mechanisms of resistance highlighting antibiotic inactivation enzymes, efflux pumps, and less frequent determinants of target modification, replacement, and protection. The dots indicate the relative abundance for each of the categories shown in the graphs, while the asterisk in (**B**) indicates the relative abundance that refers to the sum of genes that confer resistance to two or more antimicrobial classes (MDR).

A metagenomic screening was conducted on wastewater samples to identify genes potentially involved in antimicrobial resistance (Figure 1, Supplementary File S1). The analysis revealed the presence of at least 121 antimicrobial resistance genes (ARGs) in both samples, associated with resistance to 31 different types of antibacterial compounds (Figure 1A). The most abundant ARGs were linked to resistance against cephalosporins (11.6%), penams (10.76%), macrolides (9.89%), aminoglycosides (9.45%), tetracyclines (8.13%), and fluoroquinolones (7.9%). Notably, approximately 40% of the identified ARGs were associated with multidrug resistance, with around 14% conferring resistance to five or more antimicrobial classes (Figure 1B). Furthermore, the majority of detected ARGs were associated with mobile genetic elements, such as plasmids and integrons, although some were also identified on chromosomal DNA (Figure 1C). Analysis of resistance mechanisms based on gene function indicated that nearly half of the ARGs encoded antibiotic inactivation enzymes, followed by genes involved in efflux pump systems. Additional mechanisms included target modification, target replacement, and target protection (Figure 1D).

**Figure 2:**
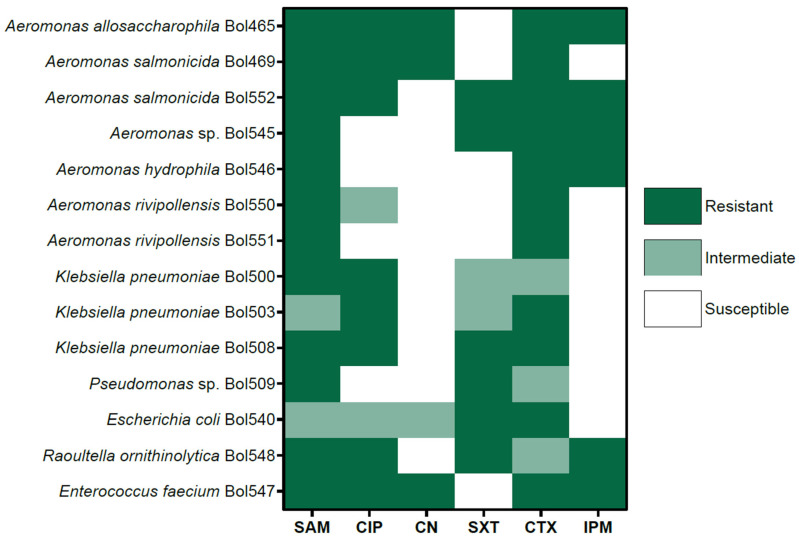
Antimicrobial susceptibility profiles of 14 bacterial isolates recovered from hospital wastewater. Isolates were tested against six antibiotics using the disk diffusion method: Ampicillin/Sulbactam (SAM), Ciprofloxacin (CIP), Gentamicin (CN), Trimethoprim/Sulfamethoxazole (SXT), Cefotaxime (CTX), and Imipenem (IPM). *Aeromonas* spp. showed the highest number of resistances, with *A. salmonicida* and *A. allosaccharophila* exhibiting strong resistance to nearly all antibiotics except one. Additional *Aeromonas* isolates were resistant mainly to SAM and CTX. *Enterococcus faecium* and *Raoultella ornithinolytica* were resistant to all tested antibiotics except SXT and CN, respectively. *Klebsiella pneumoniae* and *Escherichia coli* displayed multidrug resistance, particularly to SXT and CTX, while maintaining susceptibility to IPM. Overall, SAM and CTX exhibited reduced effectiveness across most isolates, whereas CIP and SXT resistance were primarily observed in *Klebsiella*, *Escherichia*, and *Enterococcus*. CN and IPM remained the most effective drugs, although resistance in *E. faecium* was noted.

In addition, bacterial cultures were established to evaluate antimicrobial resistance in hospital wastewater (Figure 2). The 14 bacterial isolates described above were tested in an antimicrobial susceptibility test using six antibiotics (Supplementary Table S1). Antibacterial drugs used were Ampicillin/Sulbactam, Ciprofloxacin, Gentamicin, Trimethoprim/Sulfamethoxazole, Cefotaxime, and Imipenem (namely as SAM, CIP, CN, SXT, CTX, and IPM, respectively). Among the susceptibility tests, *Aeromonas* spp. species were the strains that exhibited the lowest susceptibility to different drugs. Specifically, the isolates identified as *Aeromonas salmonicida* and *Aeromonas allosaccharophilla*, respectively, displayed strong resistance to almost all the tested antibiotics but one. Other *Aeromonas* isolates were also identified as *A. salmonicida* and *Aeromonas* spp. showed resistance mainly to Ampicillin/Sulbactam and Cefotaxime. Conversely, isolates *Enterococcus faecium* and *Raoultella ornithinolytica* were resistant to all antibiotics except Trimethoprim/Sulfamethoxazole and Gentamicin, respectively. The *Klebsiella* spp. and *Escherichia coli* isolates exhibited a loss of susceptibility to Trimethoprim/Sulfamethoxazole and Cefotaxime. The *E. coli* strain only exhibited high susceptibility to Imipenem. Overall, ampicillin/sulbactam and cefotaxime resistance were present in all the isolates, showing reduced effectiveness. Next, ciprofloxacin (CIP) and trimethoprim-sulfamethoxazole (SXT) resistances were presented in *Klebsiella*, *Escherichia*, and *Enterococcus* isolates, while *Aeromonas* isolates were susceptible. Finally, gentamicin (CN) and imipenem (IPM) exhibited higher efficacy, with fewer resistant isolates to these drugs, although *Enterococcus* spp. isolate was resistant to both antimicrobials (Figure 2). In summary, antimicrobial susceptibility testing revealed a high prevalence of resistance among the bacterial isolates recovered from hospital wastewater.

### 2.3. Physicochemical and Pharmaceutical Detection in Hospital Wastewater

**Figure 3:**
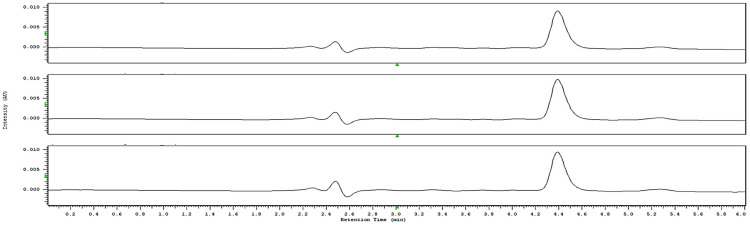
Chromatographic detection of paracetamol in hospital wastewater using HPLC-DAD. The chromatogram, shown in triplicate, reveals a well-defined peak at a retention time of approximately 4.4 min, corresponding to paracetamol. The compound was quantified at an average concentration of 277.4 ± 10.7 µg/L, with a maximum of 288.1 µg/L. In contrast, ampicillin was analyzed under the same methodology but was not detected, likely due to its rapid degradation in aqueous matrices, as also reported in previous analytical studies of wastewater effluents.

The physicochemical characterization of hospital wastewater revealed values that remained consistently below the maximum permissible limits established by Chilean regulations “Decreto 609” for effluent discharges [21]. Measured pH values ranged between 7.5 and 7.9 units, indicating circumneutral conditions well within the acceptable regulatory range. Electrical conductivity was slightly moderate (432–652 µS/cm). Total dissolved solids (TDS) varied from 218 to 328 ppm, below the regulatory limits. Chloride concentrations exceeded 200 ppm but did not surpass the regulatory limits, and reduced nitrogen forms, such as ammonium (N-NH_4_^+^), were detected at 51–73 ppm without exceeding the maximum values allowed. Phosphorus-related species (4.3–5.5 ppm) also remained within permissible levels. Taken together, these results demonstrate that the hospital wastewater studied complies with Chilean standards for physicochemical parameters, confirming that the effluent does not exceed the regulatory thresholds for discharge into sewage systems. Additionally, two commonly used drugs in Chile were also evaluated in the hospital effluent through analytical methods. Paracetamol was successfully detected (Figure 3) at an average concentration of 277.4 ± 10.7 µg/L, with a maximum value of 288 µg/L. The method showed a limit of detection (LOD) of 5.3 µg/L and a limit of quantification (LOQ) of 17.8 µg/L, within a calibration range of 22–715 µg/L and a linearity coefficient (R^2^) of 0.9922. The average recovery of the analyte was 94.1 ± 2.70%, demonstrating satisfactory analytical performance for exploratory purposes. In contrast, amoxicillin was not detected, as the compound exhibited marked instability in the wastewater matrix, likely due to its chemical structure and rapid degradation under environmental conditions. Consequently, the quantitative assessment was continued exclusively for paracetamol. No internal standards were used at this stage, as the analytical method is still under validation, and procedural blanks consisted of wastewater samples analyzed under identical experimental conditions.

## 3. Discussion

Waste management from hospitals is a growing concern due to the accumulation and detection of emerging contaminants. Hospital wastewater is a major source of these pollutants, typically rich in pharmaceuticals, pathogens, and antimicrobial resistance genes, all of which pose significant risks to public health [5,6,22]. Our results provide the first evidence of the presence of a wide diversity of emerging contaminants in hospital wastewater from southern region of Chile, highlighting the potential environmental impact exacerbated by insufficient public policies in Chile regarding wastewater treatment. It is important to acknowledge that this exploratory study was based on only two sampling campaigns conducted in August and October 2024, which may limit the representativeness of the findings. Temporal variability in hospital wastewater composition can be influenced by multiple factors, including fluctuations in patient load, variations in antibiotic prescription patterns, and hydrological dynamics associated with rainfall and temperature. These elements can affect both microbial community structure and the occurrence of chemical and genetic contaminants. Nevertheless, despite the limited number of samples, we maximized the analytical value of the dataset by applying a combination of complementary methodologies, including classical microbiological assays, chemical analyses, and shotgun metagenomic profiling, to obtain a comprehensive characterization of the hospital effluent. Consequently, the results presented here should be interpreted as an exploratory baseline that captures a partial yet informative snapshot of the system’s complexity. Future studies incorporating multi-seasonal or longitudinal sampling designs will be essential to better understand temporal trends and to improve the robustness of the conclusions derived from this research. Our findings are consistent with the strategic objectives of Chile’s “Plan Nacional Contra la Resistencia a los Antimicrobianos, Chile 2021–2022” [23], both of which emphasize the need to strengthen environmental surveillance systems and to integrate data from healthcare and environmental sectors under the One Health framework. The detection of antimicrobial resistance genes and potential pathogens in hospital effluents highlights the importance of including wastewater monitoring as a complementary tool for AMR prevention and control. By documenting the microbiological, chemical, and genomic characteristics of untreated hospital wastewater, this study contributes baseline evidence that supports national efforts to identify environmental reservoirs of resistance and to promote intersectoral collaboration for sustainable antimicrobial stewardship in Chile.

Hospital wastewater harbors clinically relevant bacteria belonging to the ESKAPEE group, including *Enterococcus faecium*, *Klebsiella pneumoniae*, *Pseudomonas aeruginosa*, and *Escherichia coli* [24]. These species are frequently found in both municipal and hospital wastewater, indicating significant pathogenic potential due to their ability to disseminate into the environment [25]. Consistent with the characteristics of this group, the isolates exhibited multidrug resistance to commonly used antibiotics such as ampicillin, ciprofloxacin, and sulfamethoxazole, which are frequently acquired within hospital-associated wastewater systems [24]. Particularly, genes identified as virulence factors were detected and associated with members of the ESKAPEE group. Most of the genes related to *Pseudomonas* encoded motility proteins linked to the flagellum (*flg*, *flh*, and *fli* genes) or type IV pili (*pil* genes), which, in addition to enabling motility, play critical roles in bacterial survival, host colonization, and pathogenicity [26]. Additionally, genes involved in alginate metabolism (*alg* genes) were found to be abundant in wastewater, potentially contributing to biofilm matrix formation and, consequently, enhancing bacterial virulence [27]. A plausible explanation for the higher abundance of *alg* genes in August sample may be related to seasonal or operational factors influencing the wastewater composition, such as variations in temperature, nutrient availability, and organic load, which could favor the proliferation of bacterial populations capable of alginate biosynthesis. Conversely, their absence in October might reflect shifts in hospital activity, wastewater flow dynamics, or competitive microbial interactions that modulate the expression or persistence of these genes in the system. Furthermore, virulence factors associated with *E. coli* were also detected. In this case, genes encoding pili were commonly observed. However, *E. coli* exhibited a broader diversity of virulence-associated genes, including those coding for enterotoxins, membrane proteins, and adhesins, probably due to its high abundance in the human gut and its well-known role as a key pathogen in common intestinal diseases [28]. Additionally, other virulence genes related to membrane transport were identified, associated with highly pathogenic bacteria such as *Salmonella*, *Neisseria*, *Shigella*, and *Brucella*. Furthermore, other medically important species were also detected in wastewater, with members of the *Aeromonas* group being particularly noteworthy due to their high diversity. These opportunistic waterborne pathogens, associated with various gastrointestinal diseases and wound infections, are commonly found in hospital settings and, consequently, in their wastewater [29]. A total of seven *Aeromonas* strains were detected through our analysis and showing high resistance to the tested antibiotic, including *Aeromonas salmonicida,* which was resistant to all the antimicrobials. Particularly, the detection of this species was not directly expected since it was described mainly as a fish pathogen; nevertheless, mesophilic strains are associated with infections in mammals, including humans, and have also been isolated from municipal and hospital wastewater [29,30,31]. However, a limitation to consider in species identification is related to the taxonomic resolution of 16S rRNA gene sequencing performed. In our dataset, some BLAST identity values were as low as 88%, which do not reliably support species-level classification. In these cases, the corresponding isolates were reassigned to the genus level to avoid overinterpretation of the data. Such variability in sequence similarity may reflect either incomplete reference coverage in public databases or the presence of uncharacterized environmental taxa with limited genomic representation. Although this limitation constrains fine-scale taxonomic resolution, the approach still provides valuable insights into the overall structure and diversity of bacterial communities present in hospital effluents. Future studies employing full-length 16S sequencing or whole-genome analyses could improve species-level discrimination and strengthen the accuracy of microbial identification.

Other bacteria of high clinical relevance due to their multidrug resistance were also detected, particularly those resistant to imipenem, a carbapenem commonly used to treat difficult-to-treat resistant (DTR) infections [32]. In this context, *Enterococcus faecium*, an emerging public health threat due to its multidrug resistance, including resistance to vancomycin, displayed a broad resistance profile, showing susceptibility only to sulfamethoxazole [33]. Reports on this member of the Enterococcaceae family have warned about its presence in regional hospitals and the rising prevalence of multidrug-resistant strains, consistent with the findings of our analysis and representing a major menace in the near future [34]. Other multiresistant DTR pathogens detected included *Klebsiella pneumoniae* isolates, which exhibited a resistant core to SAM, CIP, SXT, and CTX. This common hospital-associated pathogen has developed and acquired multiple resistance mechanisms such as β-lactamases, efflux pumps, and others, which, together with its biofilm-forming ability, make it a major threat due to its association with high morbidity and mortality rates [32,35,36,37]. Finally, *Raoultella ornithinolytica* was isolated from the wastewater samples. Although it is more commonly associated with environmental sources and remains an uncommon bacterium in clinical studies, it has been linked to bacteremia and other human infections (for example, urinary infections) [38]. Previous reports showed a good response of antimicrobial agents to its infections, and a low susceptibility to ampicillin, piperacillin, and fosfomycin [39]. In concordance, our microbial isolate was resistant to ampicillin as well as to CIP, SXT, and IMP drugs.

Metagenomic screening revealed concordance between the resistance genes detected and the results of the antimicrobial susceptibility tests. In particular, genes associated with resistance to fluoroquinolones (ciprofloxacin), aminoglycosides (gentamicin), cephalosporins (cefotaxime), and penicillin-related antibiotics (ampicillin) were identified. In general, these classes of antimicrobials, and their corresponding resistance genes, are commonly found in wastewater [31,40] and in the environment [41,42]. The persistence of these resistance genes may result from the persistence of antimicrobial compounds in hospital effluents. However, it has been demonstrated that direct treatment of hospital wastewater can efficiently remove antimicrobials and therefore represents a necessary intervention to reduce the environmental spread of resistance [43]. Other major resistance genes not evaluated by susceptibility testing but detected through metagenomic analysis included those conferring resistance to macrolides, penams, and tetracyclines, which are frequent targets of resistance, particularly in hospital wastewater, where these antibiotics are among the most widely used [44,45]. Antimicrobial resistance genes also revealed trends regarding their nature in wastewater. As expected, a high proportion of the detected genes conferred human-use drug resistance, which may be influenced by hospital-related factors such as infrastructure, waste handling practices, antibiotic usage, and disposal patterns [4,5,31,43]. Moreover, most of the detected genes were associated with mobile genetic elements such as plasmids and integrons, which raises significant concern about the potential spread of resistance among environmental microorganisms [41,42,46,47].

The presence of pharmaceutical compounds was also evaluated in the wastewater, with a focus on two of the most used pharmaceutical compounds in Chile: paracetamol and amoxicillin. Paracetamol is often the most abundant analgesic detected in hospital wastewater. Previous studies have reported paracetamol concentrations in wastewater ranging from 41 to 210 µg/L, although levels as high as 675 µg/L have also been documented [48,49]. These findings suggest that paracetamol use is widespread in southern Chile, which may pose environmental risks to aquatic ecosystems [50,51]. On the other hand, amoxicillin (a widely used penicillin-derived antibiotic) was not detected in our analysis. To date, measurements of amoxicillin in hospital wastewater remain limited, but reported concentrations vary significantly by region, ranging from 0.11 µg/L in France to 11,720 µg/L in India [52]. In this study, the absence of amoxicillin detection can be attributed to a combination of analytical limitations and the compound’s inherent instability in wastewater. As a β-lactam antibiotic, amoxicillin undergoes rapid hydrolysis, which markedly reduces its persistence in aquatic environments. Moreover, matrix effects may further compromise its recovery during HPLC analysis. These factors together indicate that, despite its widespread clinical use, the environmental impact of amoxicillin is likely underestimated due to its low stability and high reactivity in complex effluent systems. This does not exclude the presence of other pharmaceutical compounds in hospital wastewater, which may contribute to the global challenge of antimicrobial resistance.

These findings demonstrate that the measured pharmaceutical concentrations, together with the detection of antimicrobial resistance and virulence genes in untreated hospital wastewater, pose potential risks to both environmental and human health. Such contaminants can be transported through sewer systems, entering common wastewater treatment plants, where horizontal gene transfer and bacterial proliferation may occur, and eventually reaching receiving water bodies if removal is incomplete [53]. This scenario underscores the relevance of exposure pathways connecting hospital effluents with downstream receptors such as aquatic ecosystems and communities relying on these resources. To mitigate these risks, implementing advanced treatment technologies (such as ozonation, advanced oxidation processes (AOP), UV-AOP hybrids, and membrane bioreactors) is essential, as these methods have demonstrated high efficacy (>80–95%) in degrading pharmaceuticals and eliminating ARGs and pathogens in similar effluent matrices [54,55]. Strengthening hospital wastewater management through such technologies, along with systematic monitoring programs, will be crucial to reduce the dissemination of emerging contaminants and safeguard public and environmental health in Chile and beyond. Therefore, these findings emphasize the importance of updating regulations and decrees governing wastewater discharges, ensuring that detection methodologies and permissible thresholds adequately address the presence of emerging pharmaceutical contaminants and their contribution to the persistence of antimicrobial resistance in the environment.

## 4. Materials and Methods

### 4.1. Wastewater Sample Collection and Microbial Isolation

A total of 5 L of raw wastewater was collected during two independent sampling campaigns conducted in August and October 2024 from the main sewer pipeline of the hospital (Supplementary Figure S1). Samples were obtained using pre-sterilized glass bottles, handled with aseptic technique to avoid cross-contamination. Immediately after collection, the bottles were placed in insulated containers with ice packs to maintain a temperature of 4 °C and transported within one hour to the Patagonia Institutional Laboratory at Universidad San Sebastián (Puerto Montt, Chile). All samples were processed on the same day of collection to minimize physicochemical alterations and microbial shifts during storage. For microbiological and molecular analyses, wastewater was processed through vacuum-assisted filtration using a glass filtration system (Merck-Millipore, Burlington, MA, USA) equipped with sterile mixed cellulose ester (MCE) membranes of 0.22 µm pore size (Merck-Millipore, USA). The retained biomass on the filters was subsequently preserved in RNA Later solution (Sigma-Aldrich, St. Louis, MO, USA) and stored at −20 °C until further DNA extraction as described below. This approach ensured the capture of both planktonic bacteria and extracellular genetic material present in the effluent. In parallel, aliquots of unfiltered effluents were serially diluted and streaked on different culture media, including CHROMagar™ Orientation (CHROMagar, Saint-Denis, France), Brain Heart Infusion agar (Becton Dickinson, Franklin Lakes, NJ, USA), Tryptic Soy agar (Becton Dickinson, USA), MacConkey agar (Becton Dickinson, USA), Eosin Methylene Blue agar (Becton Dickinson, USA), and Mueller-Hinton agar (BD DIFCO, Wokingham, UK), to recover a wide spectrum of bacterial taxa. Inoculated plates were incubated at 25 °C and 37 °C for 24–48 h to promote the growth of both mesophilic and environmental isolates. Single colonies displaying distinct morphological traits were repeatedly sub-cultured onto fresh plates of the same medium to achieve pure cultures. Purity was confirmed by Gram staining and microscopic observation. Pure isolates were preserved in cryovials containing 10% (*v*/*v*) sterile glycerol and stored at −80 °C for long-term maintenance. All procedures involving potentially multidrug-resistant (MDR) isolates were conducted under biosafety level 2 (BSL-2) conditions, following institutional guidelines. Cultures and contaminated materials were autoclaved and disposed of according to standard biosafety and environmental safety protocols to prevent any accidental release or cross-contamination.

### 4.2. Microbial Characterization

The taxonomic identification of bacterial isolates obtained from hospital wastewater was performed through a combination of phenotypic and molecular approaches. Preliminary identification included the observation of colony morphology on selective media and Gram staining to differentiate Gram-positive and Gram-negative bacteria, providing initial insight into the dominant microbial groups present in the effluent. Subsequently, molecular identification was carried out by amplification of the 16S rRNA gene using the universal primers 27F (5′-AGAGTTTGATCMTGGCTCAG-3′) and 1492R (5′-GGTTACCTTGTTACGACTT-3′), as originally described [56]. The amplicons, approximately 1500 base pairs in length, were verified by electrophoresis and subsequently purified for sequencing using the Sanger method by means of MACROGENE (Seoul, Korea) sequencing service. The resulting sequences were compared against reference sequences in the NCBI BLASTn database [57] to determine the closest taxonomic affiliation based on sequence similarity and E-values, using the 16s ribosomal RNA sequence (bacteria and archea) database from rRNA/ITS database category. This combined phenotypic–genotypic approach enabled accurate taxonomic assignment of environmental isolates, allowing the identification of several genera of clinical and environmental relevance within hospital effluents.

### 4.3. Antimicrobial Susceptibility Assay

Antimicrobial susceptibility testing was performed in triplicate on the 14 bacterial isolates recovered and identified from hospital wastewater using the standardized disk diffusion method (Kirby–Bauer), following the protocol described by Hudzicki (2009) and adapted from the Clinical and Laboratory Standards Institute (CLSI) guidelines [58,59]. To ensure that the isolates were in logarithmic growth phase, subcultures were prepared for three consecutive days prior to the assay. From each subculture plate, a loopful of bacterial biomass was transferred into sterile test tubes containing 2 mL of distilled water and homogenized for 20 s by vortex. The turbidity of each suspension was adjusted to match a 0.5 McFarland standard, either visually or with the aid of a turbidimeter, to ensure a standardized inoculum density equivalent to approximately 1.5 × 10^8^ CFU/mL. Using sterile cotton swabs, each suspension was spread uniformly over the surface of Mueller-Hinton agar plates (BD DIFCO, Franklin Lakes, NJ, USA) by rotating the plate 60° between streaking to achieve a confluent lawn of growth. After allowing the excess surface moisture to evaporate for 1 min under sterile conditions, antibiotic-impregnated disks were placed on the agar surface using flame-sterilized and cooled forceps. The panel of antimicrobials used (Oxoid, Hampshire, UK) included cefotaxime (CTX, 30 µg), ampicillin/sulbactam (SAM, 10/10 µg), sulfamethoxazole/trimethoprim (SXT, 1.25/23.75 µg), ciprofloxacin (CIP, 5 µg), imipenem (IPM, 10 µg), and gentamicin (CN, 10 µg). These compounds were chosen to represent different major classes of antibiotics commonly used in Chilean clinical settings, including a synthetic penicillin combined with a β-lactamase inhibitor, a cephalosporin, a carbapenem, a fluoroquinolone, an aminoglycoside, and a combination of folate pathway inhibitors. The selection was guided by their clinical relevance and prescription frequency in Chilean hospitals, as reported by the Chilean “Plan Nacional Contra la Resistencia a los Antimicrobianos, Chile 2021–2022” [23] and consistent with the WHO AWaRe classification of essential antimicrobials. This panel therefore provides a representative overview of therapeutic agents that are widely employed in both empirical and targeted treatments, allowing a meaningful assessment of antimicrobial resistance patterns in hospital wastewater isolates.

Disks were distributed evenly on the plate surface, maintaining a minimum distance of 30 mm between disk centers to avoid overlapping inhibition zones. Plates were incubated at 37 °C for 24 h in aerobic conditions. After incubation, the diameter of each inhibition zone was measured in millimeters using a metric ruler. Each assay was performed in triplicate, and results were expressed as the mean ± standard deviation. Zone diameters were interpreted according to CLSI breakpoint criteria [59]. For quality control, *Escherichia coli* ATCC^®^ 25922 was included in all assays to validate the performance of the method [58,59]. Biological residues generated during the procedure were disinfected by immersion in a sodium hypochlorite 1% solution for at least 10 min and subsequently disposed of following biosafety guidelines established for clinical microbiology laboratories.

### 4.4. DNA Isolation, Shotgun Sequencing, and Metagenomic Analysis

Total genomic DNA was extracted from the biomass retained on mixed cellulose ester (MCE) filters previously stored in RNA Later solution. Extraction was carried out using the AccuPrep Genomic DNA Extraction Kit (Bioneer, Daejeon, Republic of Korea), following the manufacturer’s recommendations with slight modifications to improve lysis efficiency. The modified protocol has been previously applied in other metagenomic studies investigating microbial communities in aquatic environments [41,42,60]. Briefly, each filter was submerged in 500 µL of lysis buffer and mechanically agitated to release microbial cells from the membrane surface. To ensure effective cell disruption, an enzymatic cocktail consisting of 20 µL of lysozyme (20 mg/mL) and 20 µL of proteinase K (20 mg/mL) was added. The suspensions were incubated sequentially for 1 h at 37 °C to facilitate lysozyme activity, followed by 1 h at 55 °C to complete protein digestion. Subsequent DNA purification steps, including binding, washing, and elution, were performed according to the kit protocol, yielding highly concentrated DNA suitable for downstream applications. DNA quality and integrity were evaluated by electrophoresis on 1% agarose gels, while DNA concentration and purity were assessed spectrophotometrically by measuring absorbance at 260 nm and calculating the 260/280 ratios. Additionally, PCR amplification of the bacterial 16S rRNA gene using universal primers 27F and 1492R [56] was performed to validate the suitability of the extracted DNA for metagenomic sequencing. A total of 1 µg of purified DNA per sample was sent to Novogene (Sacramento, CA, USA) for whole-metagenome shotgun sequencing. Libraries were prepared and sequenced on an Illumina NovaSeq 6000 platform (San Diego, CA, USA), generating paired-end reads (2 × 150 bp) with an average yield of 6 GB per sample. Bioinformatic analysis was performed to ensure data quality and enable comprehensive functional and taxonomic profiling. Raw reads were first inspected with FastQC [61] to assess overall quality parameters. Filtering and trimming were conducted using Trimmomatic [62], using the parameters: LEADING:20, TRAILING:20, SLIDINGWINDOW:5:20, AVGQUAL:20, MINLEN:90, and contaminant sequences from humans and viruses were removed by mapping against reference genomes with Bowtie2 [63]. The cleaned reads were assembled de novo using MEGAHIT [64], and assembly metrics were evaluated with QUAST [65]. Taxonomic classification was carried out using Kraken2 [66,67] using the Standard with DB capped at 16 GB database from 4 February 2025, retaining taxonomic assignments with more than 50 hits per sample, and relative abundances were calculated at the species level. Functional annotation was focused on the detection of antimicrobial resistance and virulence determinants. Assembled contigs were screened with ABRicate v1.0.1 [68] against the CARD v4.0.1 [69] and VFDB update 2025 database [70,71]. All processed data were imported into the R statistical language [72] for downstream analysis and visualization using the ggplot2 [73] package.

### 4.5. Physicochemical Analysis of Wastewater

Physicochemical parameters of hospital effluents were determined following standardized procedures to evaluate compliance with the “Decreto 609” Chilean water quality regulations [21]. Measurements included pH, electrical conductivity (EC), total dissolved solids (TDS), chloride ions, phosphorus, and nitrogen-related compounds, which were analyzed using potentiometric and spectrophotometric methods. Samples were collected and processed immediately to minimize alterations, and all analyses were performed in triplicate to ensure reproducibility.

### 4.6. Pharmaceutical Analysis by HPLC-DAD

Wastewater samples were processed in triplicate for the detection of paracetamol and amoxicillin using high-performance liquid chromatography with diode array detection (HPLC-DAD). For this purpose, a new methodology was developed for the simultaneous detection of both molecules. Briefly, 1 L of each sample was collected in amber glass bottles and stored at 4 °C until analysis. Pre-treatment involved sonication (5 min), centrifugation (4000 rpm, 10 min), and sequential filtration through PVDF membranes (0.45 and 0.22 µm) to remove suspended solids. For preconcentration and clean-up, 100 mL of filtrate were subjected to solid-phase extraction (SPE) using Supel™ Swift HLB cartridges (60 mg, 3 mL, Merck, Darmstadt, Germany), previously conditioned with methanol and ultrapure water. After sample loading, cartridges were washed with ultrapure water, dried under controlled vacuum, and analytes were eluted with 4 mL of methanol. Eluates were diluted in the corresponding mobile phase before chromatographic analysis. Calibration curves were prepared from standard solutions of paracetamol (USP grade) and amoxicillin trihydrate (Merck) in the range of 22–715 µg/L, yielding correlation coefficients of 0.99. Chromatographic separation was performed on a Nucleosil^®^ C18 column (250 mm × 3.2 mm, 5 µm; Supelco, Bellefonte, PA, USA) at 35 °C, using a gradient of ultrapure water (phase A) and methanol (phase B) at a flow rate of 1.0 mL/min. Detection was carried out at 243 nm with injection volumes of 90 µL. The standard retention times were approximately 2.93 min for amoxicillin (Sigma-Aldrich, USA) and 4.35 min for paracetamol (Merck, USA), respectively. The HPLC-DAD method demonstrated high analytical precision, with recovery rates above 90% for paracetamol and acceptable reproducibility for amoxicillin, confirming the reliability of the procedure for quantifying pharmaceutical residues in wastewater samples.

## 5. Conclusions

This study provides the first comprehensive characterization of hospital wastewater in southern Chile, integrating physicochemical, microbiological, and metagenomic approaches. The effluent exhibited circumneutral to slightly alkaline conditions, moderate conductivity, and elevated levels of nitrogen compounds and chlorides, together with detectable concentrations of paracetamol. Microbial culture and sequencing revealed the presence of clinically relevant pathogens, particularly members of the ESKAPEE group and *Aeromonas* spp., many of which displayed multidrug resistance phenotypes. Notably, resistance to β-lactams, fluoroquinolones, and sulfonamides was widespread, with *Enterococcus faecium* and *Klebsiella pneumoniae* representing the most concerning isolates due to their broad resistance patterns. Metagenomic screening corroborated these findings, identifying more than 120 antimicrobial resistance genes, many associated with mobile genetic elements, multidrug resistance, and virulence determinants. Taken together, these results demonstrate that, although the physicochemical composition of hospital effluents complies with current Chilean regulatory standards, it nonetheless constitutes a critical reservoir of antimicrobial resistance and emerging contaminants. This dual condition highlights the hidden risk that such effluents pose, with the potential to disseminate resistant bacteria and genes into surrounding ecosystems. The findings underscore the urgent need for systematic monitoring, the implementation of advanced wastewater treatment technologies, and strengthened public health policies to mitigate the environmental spread of antimicrobial resistance.

## Figures and Tables

**Table 1:** Bacterial isolates recovered from hospital wastewater. Identification was performed by 16s rRNA PCR and BLAST Version 2.17.0 sequence alignment, showing percentage identity with reference strains. Each isolate was assigned a strain code within the institutional culture collection. The dataset reflects the predominance of Gram-negative bacteria, particularly members of the genus *Aeromonas*, along with clinically relevant genus such as *Escherichia*, *Klebsiella*, *Pseudomonas*, and *Raoultella*. One Gram-positive isolate, *Enterococcus*, was also identified, underscoring the heterogeneous and clinically significant composition of the effluent microbiota. Each isolate was assigned a strain code (BOL), which corresponds to the internal culture collection of our laboratory.

Microbial Genus	BLAST Identity	Strain Number
*Aeromonas*	93.3%	BOL465
*Aeromonas*	91.6%	BOL546
*Aeromonas*	89.8%	BOL550
*Aeromonas*	88.1%	BOL551
*Aeromonas*	89.1%	BOL469
*Aeromonas*	93.9%	BOL552
*Aeromonas.*	90.3%	BOL545
*Enterococcus*	94.6%	BOL547
*Escherichia*	91.8%	BOL540
*Klebsiella*	96.3%	BOL500
*Klebsiella*	94.4%	BOL503
*Klebsiella*	95.0%	BOL508
*Pseudomonas.*	91.6%	BOL509
*Raoultella*	96.5%	BOL548

**Table 2:** High abundance Virulence-associated gene families detected in hospital wastewater metagenomes. Genes were identified through screening against the VFDB database and classified by family, subtype, host bacteria, and functional description. The majority of the identified genes were linked to motility and biofilm formation, including the *flg*, *fli*, *pil*, and *alg* families. Notably, *Pseudomonas aeruginosa* carried the highest number of virulence-related genes, while additional determinants were found in *Aeromonas hydrophila*, *A. salmonicida*, *Acinetobacter baumannii*, and *Klebsiella pneumoniae*. Less frequently, virulence markers associated with *Escherichia coli*, *Salmonella enterica*, *Neisseria meningitidis*, *Brucella melitensis*, and *Shigella dysenteriae* were also detected, underscoring the heterogeneous pathogenic potential of the effluent microbiome.

Gene Family	Subtype	Bacteria	Description
*alg*	C, R, W, U, Z	*P. aeruginosa*	Biofilm formation, flagellar biogenesis, motility
*fle*	N, Q	*P. aeruginosa*, *A. hydrophyla*	Biofilm formation, flagellar biogenesis, motility
*flg*	C, G, H, L	*P. aeruginosa*, *A. hydrophyla*, *A. salmonicida*	Biofilm formation, flagellar biogenesis, motility
*flh*	A	*P. aeruginosa*, *A. hydrophyla*, *A. salmonicida*	Flagellar biogenesis
*fli*	A, E, G, I, M, P, Q	*P. aeruginosa*, *A. hydrophyla*, *A. salmonicida*	Flagellar assembly and biogenesis
*mot*	A	*A. hydrophyla*, *A. salmonicida*	Bacterial flagelar motility
*pil*	G, H, J, M, R, T, U	*A. baumannii*, *P. aeruginosa*	Pili biogenesis regulation
*ykg*	K	*K. pneumoniae*	Transcriptional regulator
*ent*	A, B	*K. pneumoniae*	Iron uptake
*gsp*	G, H, J, M, R, T, U	*A. baumannii*, *K. pneumoniae*	Extracellular transport
*omp*	A	*A. baumannii*, *A. hydrophyla*, *A. salmonicida*, *K. pneumoniae*	Adhesion, pore formation, immune evasion

## Data Availability

The raw data produced from DNA sequencing in this study were deposited in the ENA-EMBL database under the accession number PRJEB96307 (https://www.ebi.ac.uk/ena/browser/view/PRJEB96307, accessed on 23 August 2025). Metagenomic data obtained from bioinformatics analysis can be found in Supplementary Files.

## Supplementary Materials

The following supporting information can be downloaded at: https://www.mdpi.com/article/10.3390/antibiotics14111111/s1, Supplementary File S1. This file contains the taxonomic profiles generated with Kraken2, representing the relative abundance of microbial taxa in wastewater samples collected in August (Sample 1) and October (Sample 2) 2024. It also includes the antimicrobial resistance (AMR) genes identified with ABRicate using the CARD database, and virulence-associated genes detected with VFDB. For both AMR and virulence genes datasets, the reported numbers correspond to the percentage of gene coverage, while repeated entries indicate the presence of multiple copies of the same gene within a sample. Supplementary Table S1. Antimicrobial susceptibility profiles of bacterial isolates recovered from hospital wastewater. The table reports inhibition zone diameters (IZD, expressed in millimeters ± standard deviation) obtained by disk diffusion assays, along with the categorical interpretation (S: susceptible; I: intermediate; R: resistant) based on CLSI breakpoints. The tested antibiotics were Ampicillin/Sulbactam (SAM), Ciprofloxacin (CIP), Gentamicin (CN), Trimethoprim/Sulfamethoxazole (SXT), Cefotaxime (CTX), and Imipenem (IPM). Supplementary Figure S1. Schematic representation and photographic documentation of the sampling site for hospital wastewater collection.
