# Supplementary material for: Hospital Wastewater as a Reservoir of Contaminants of Emerging Concern: A Study Report from South America, Chile
Source: Antibiotics (Basel). 2025 Nov 4;14(11):1111. doi: 10.3390/antibiotics14111111 (PMC12649306; doi:10.3390/antibiotics14111111)
Supplement: Supplementary file 1 [file antibiotics-14-01111-s001.zip › Supplementary Figure1.pdf]

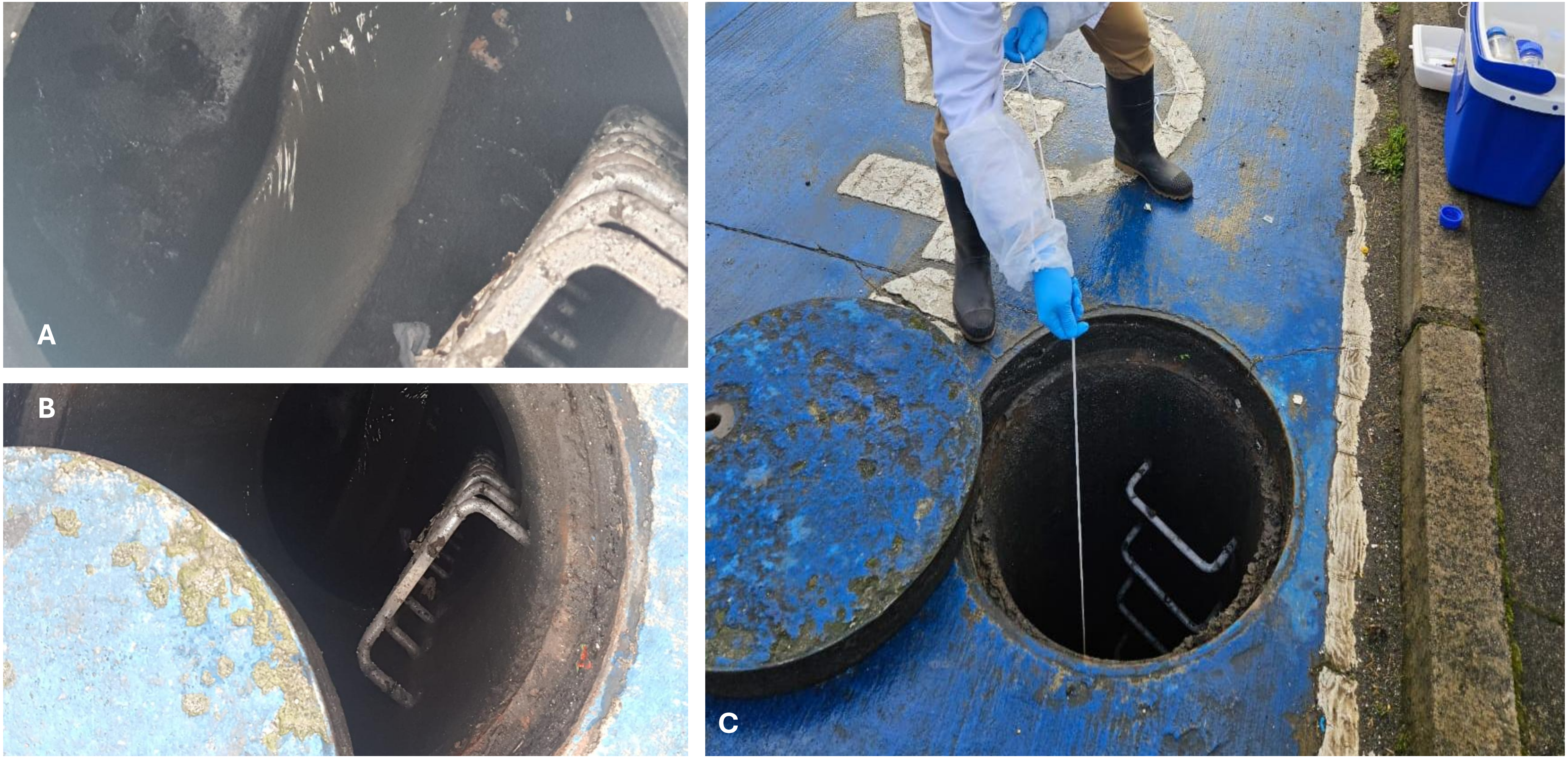

Figure Supplementary 1. Schematic representation and photographic documentation of the sampling site for hospital wastewater collection. (A) Sewer line layout illustrating the location of the hospital discharge point prior to connection with the municipal wastewater network. (B) Inspection cover of the drainage system, indicating the access point used for sampling. (C) On-site collection of wastewater samples directly from the drainage outlet using sterile equipment. At this point, all internal discharges from the hospital converge and flow toward the public sewer system, representing the main outlet of untreated effluent. This location corresponds to the sampling site used for the microbiological, chemical, and metagenomic analyses described in this study.
