# Supplementary material for: Hospital Wastewater as a Reservoir of Contaminants of Emerging Concern: A Study Report from South America, Chile
Source: Antibiotics (Basel). 2025 Nov 4;14(11):1111. doi: 10.3390/antibiotics14111111 (PMC12649306; doi:10.3390/antibiotics14111111)
Supplement: Supplementary file 1 [file antibiotics-14-01111-s001.zip › Supplementary Table1.pdf]

BOL N°	Species Identified	Antimicrobial Agents Used											
		Ampicillin/Sulbactam (SAM)		*Ciprofloxacin* (CIP)		*Gentamicin* (CN)		*Trimethoprim/Sulfamethoxazole* (STX)		*Cefotaxime* (CTX)		*Imipenem* (IPM)	
		IZD (mm)	Int	IZD (mm)	Int	IZD (mm)	Int	IZD (mm)	Int	IZD (mm)	Int	IZD (mm)	Int
465	*Aeromonas allosaccharophila*	6± 0,6	IR	9± 0,6	R	6±0,0	R	24±2,1	S	6± 0,6	R	17±2,0	R
469	*Aeromonas salmonicida*	8± 1,0	IR	13±3,2	R	8±0,6	R	21±1,0	S	10±1,0	R	36±2,1	S
500	*Klebsiella pneumoniae*	11 ±0,0	R	14±3,5	R	20±1,2	S	13±1,0	I	7 ± 0,6	R	24±3,1	S
503	*Klebsiella pneumoniae*	12±1,5	I	17 ±1,0	R	22±1,5	S	15±0,6	I	7± 1,0	R	24±1,0	S
508	*Klebsiella pneumoniae*	8±1,5	R	7±0,0	R	20±1,0	S	7±0,6	R	7±0,0	R	26±1,2	S
509	*Pseudomonas* spp.	8 ± 1,7	IR	43±0,6	IE	26±2,3	IE	6±0,0	IR	23±2,0	IR	40±0,6	IE
540	*Escherichia coli*	13±1,5	I	24±4,0	I	15±0,6	I	7±0,0	R	7,7±0,6	R	23±1,0	S
545	*Aeromonas spp.*	6±0,6	IR	28±1,0	S	27±1,2	S	7±0,0	R	14±1,2	R	13±2,9	R
546	*Aeromonas hydrophila*	7±0,6	IR	26±1,5	S	23±4,0	S	27±1,0	S	8±3,2	R	19±0,6	R
547	*Enterococcus faecium*	6±0,6	NA	6±0,6	R	6±0,0	NA	29±0.6	NA	6±0,6	IR	7±0,6	IR
548	*Raoultella ornithinolytica*	7±0,6	R	17±2,5	R	20±1,5	S	6±0,6	R	23±1,5	I	16±3,0	R
550	*Aeromonas rivipollensis*	7±0,6	IR	22±2,6	S	22±1,7	S	16±0,6	S	10±3,0	R	35±3,5	S
551	Aeromonas rivipollensis	10± 1,5	IR	25±1,5	S	24± 1,7	S	16±3,6	S	7±4,0	R	37±2,1	S
552	*Aeromonas salmonicida*	7±0,0	IR	6±1,0	R	23±3,8	S	6±1,0	R	6±1,0	R	10±3,0	R
ATCC	*Escherichia coli*	20±2,0	✓	31± 3,4	✓	22±2,5	✓	26±3,9	✓	31±3,4	✓	28±2,3	✓

Note. IZD: Inhibition zone diameter expressed in millimeters; ± standard deviation; Int: Interpretation according to CLSI breakpoints. S: susceptible, I: intermediate, R: resistant; IR: Intrinsic resistance (this species is intrinsically resistant to the agent. Results are not categorized as S/I/R per CLSI/EUCAST recommendations); IE: Insufficient evidence of species-specific breakpoints; NA: Not applicable; ✓: Quality Control, according to values defined by CLSI. Clinical and Laboratory Standards Institute (2024). BOL N° corresponds to the internal culture collection of our laboratory.
